# 4G/5G Variant of Plasminogen Activator Inhibitor-1 Gene and Severe Pregnancy-Induced Hypertension: Subgroup Analyses of Variants of Angiotensinogen and Endothelial Nitric Oxide Synthase

**DOI:** 10.2188/jea.JE20090003

**Published:** 2009-11-05

**Authors:** Gen Kobashi, Kaori Ohta, Hideto Yamada, Akira Hata, Hisanori Minakami, Noriaki Sakuragi, Hiko Tamashiro, Seiichiro Fujimoto, the Hokkaido Perinatal Epidemiology Study Group

**Affiliations:** 1 Molecular Biostatistics Research Team, Research Center for Charged Particle Therapy, National Institute of Radiological Science, Chiba, Japan; 2 Department of Global Health and Epidemiology, Hokkaido University Graduate School of Medicine, Sapporo, Japan; 3 Department of Obstetrics and Gynecology, Hokkaido University Graduate School of Medicine, Sapporo, Japan; 4 Department of Public Health, Chiba University Graduate School of Medicine, Chiba, Japan; 5 Sapporo Maternity Women’s Hospital, Sapporo, Japan

**Keywords:** pregnancy-induced hypertension, plasminogen activator inhibitor-1, angiotensinogen, endothelial nitric oxide synthase, gene polymorphism

## Abstract

**Background:**

Pregnancy-induced hypertension (PIH) is a common cause of perinatal mortality. It is believed to result from the interaction of several factors, including those related to the blood coagulation system. We performed genotyping and subgroup analyses to determine if the 4G/5G genotypes of the plasminogen activator inhibitor-1 gene (*PAI-1*) play a role in the pathogenesis of PIH, and to evaluate possible interactions of the *PAI-1* polymorphisms with those of the angiotensinogen gene (*AGT*) and the endothelial nitric oxide synthase gene (*NOS3*).

**Methods:**

An association study of *PAI-1* polymorphism, and subgroup analyses of common variants of *AGT* and *NOS3*, among 128 patients with PIH and 376 healthy pregnant controls.

**Results:**

No significant differences were found between the cases and controls in the frequencies of allele 4G or the 4G/4G genotype. In subgroup analyses, after adjustment for multiple comparison, a significant association with the *AGT* TT genotype was found among women with the *PAI-1* 4G/4G genotype, and an association with the *NOS3* GA+AA genotype was found among women with the 5G/5G or 4G/5G genotypes.

**Conclusions:**

Our findings suggest that there are at least 2 pathways in the pathogenesis of severe PIH. However, with respect to early prediction and prevention of severe PIH, although the *PAI-1* 4G/4G genotype alone was not a risk factor for severe PIH, the fact that *PAI-1* genotypes are associated with varying risks for severe PIH suggests that *PAI-1* genotyping of pregnant women, in combination with other tests, may be useful in the development of individualized measures that may prevent severe PIH.

## INTRODUCTION

Pregnancy-induced hypertension (PIH) is a common cause of perinatal mortality, and is believed to result from both genetic and environmental factors. PIH can be triggered by placental ischemia, which is caused by an interaction between genetic and environmental factors. Several such factors have been investigated, including pressor/depressor mechanisms, the blood coagulation system, and lipid metabolism.^1^^,^^2^ Recent advances in molecular biology and epidemiology, including the examination of a number of genetic variants, are expected to improve our understanding of the role of genetic factors in the complex etiology of PIH.^3^^–^^5^

Plasminogen activator inhibitor-1 (PAI-1) is a major inhibitor of fibrinolysis. A principal step in the fibrinolytic process is the conversion of plasminogen to plasmin, which is regulated by activators such as tissue-type plasminogen activator (t-PA) and urokinase-type plasminogen activator (u-PA). PAI-1 is a fast-acting inhibitor of t-PA and it regulates the rate of clot dissolution.^6^ When compared to normal pregnant women, higher levels of plasma and placental PAI-1 are present in women with severe preeclampsia (PE)—a proteinuric PIH.^7^ In addition, a decrease in plasma fibrinolytic activity due to increased PAI-1 levels has been reported in PE.^8^^,^^9^ A common single-nucleotide polymorphism (SNP) of the PAI-1 gene (*PAI-1*)—a guanine insertion/deletion (4G/5G polymorphism)—is located 675 base pairs upstream from the transcriptional start site in the promoter region. It has been reported that individuals with the 4G homozygote (4G/4G genotype) of *PAI-1* have a plasma PAI-1 level that is approximately 20% higher than that of individuals with other genotypes.^10^^,^^11^ To date, an association between the *PAI-1* 4G/4G genotype and PE has not been confirmed, although the possibility of such an association has been investigated.^12^^,^^13^

Regarding other genetic variants, it has been reported that T235 of the angiotensinogen gene (*AGT*) and Asp298 of the endothelial nitric oxide synthase gene (*NOS3*) are associated with PIH in pregnant Japanese women,^14^^–^^17^ although the associations were not confirmed in all studies.^3^^,^^18^^–^^20^ In a previous study using multivariate analysis, we found that *AGT* T235 and *NOS3* Asp298 were independently associated with PIH, which suggests that these 2 variants are involved in different pathways in the pathogenesis of PIH.^17^

In the present study, in order to clarify the roles of the 4G/5G variant of *PAI-1* for PIH, and to evaluate possible synergisms of the *PAI-1* variant as well as variants of *AGT* and *NOS3* in the manifestation of PIH, we carried out further genotyping to expand upon the findings of our previous association study.^17^

## METHODS

PIH cases and controls were recruited from women who delivered singletons at Hokkaido University Hospital or its affiliated hospitals. We excluded women with hemolysis, liver dysfunction, and low platelets (HELLP) syndrome; renal disease; diabetes mellitus; amniotic volume abnormalities; preexisting hypertension; or fetal anomalies. Severe PIH was diagnosed according to the criteria of the National High Blood Pressure Education Program Working Group,^21^ ie, blood pressure ≥160/110 mm Hg after the 20th gestational week, regardless of the presence of proteinurea. Women with a blood pressure ≥140/90 mm Hg or proteinurea either before the 20th gestational week or 4 weeks after delivery were excluded because of the possibility that they may have had latent hypertension or renal disease. Ultimately, 128 patients with severe PIH agreed to participate in this study between 1993 and 1997, and 376 controls were randomly selected from normal pregnant women who also agreed to participate in the study during the same time period. Informed consent for the study was obtained from every subject, and the present study was approved by the institutional review board of Hokkaido University School of Medicine.

Genomic DNA was extracted from peripheral leukocytes. Genotyping of the *PAI-1* 4G/5G, *AGT* M235T, and *NOS3* Glu298Asp polymorphisms was performed by using the polymerase chain reaction-restricted fragment length polymorphism (PCR-RFLP) method, in which the PCR products were digested with the restriction enzymes B*sl*I, T*th*111I, and B*an*II, respectively, as previously described.^22^^,^^23^ The the digested fragments were checked by electrophoresis on a 10% polyacrylamide gel. The genotyping was verified either by retesting or by use of other methods.^15^^,^^17^

Statistical analysis was performed to compare the number of the 4G alleles for *PAI-1*, and according to a comparison of the number of women possessing 4G/4G with that of women possessing the 5G homozygote (5G/5G) and the heterozygote (4G/5G) for the *PAI-1* genotype. For the *AGT* genotype, the number of T235 homozygote (TT) women was compared with that of the M235 homozygote (MM) and heterozygote (MT) women. For the *NOS3* genotype, the number of Glu298 homozygote (GG) women was compared with that of the Asp298 homozygote (AA) and heterozygote (GA) women. Furthermore, we performed subgroup analyses of the *AGT* genotype and the *NOS3* genotype in severe PIH and controls, according to the binary *PAI-1* genotypes. Differences were analyzed by using the chi-square test (degree of freedom = 1). Fisher’s exact test was used when an observed number was 5 or lower. A *P* value less than 0.05 was considered to indicate statistical significance. In the subgroup analysis, *P* values less than 0.0125 were considered to indicate statistical significance, after use of the Bonferroni correction for multiple comparison to reduce the probability of an alpha-error. In subsequent multivariate analysis, screening was performed using a stepwise method for items demonstrating a statistically significant difference, as well as product terms of the factors of the genetic variants to evaluate their interactions. All statistical analyses were conducted with the statistical analysis software SAS version 9.1 (SAS Institute Japan Ltd, Tokyo, Japan).

## RESULTS

The clinical characteristics and pregnancy outcomes of the 128 cases and 376 controls are shown in Table 1. The rate of a positive family history (FH) of hypertension and pre-pregnancy body mass index (BMI) were significantly higher among cases; there was no significant difference in maternal age or the rates of primigravid and primiparous women between the 2 groups. Number of gestational weeks at delivery and birth weight were lower among the cases. In approximately 30% of the cases, severe PIH was complicated by intrauterine growth retardation.

**Table 1.** Clinical characteristics and pregnancy outcomes of women with severe pregnancy-induced hypertension (cases) and controls

	Cases (*n* = 128)	Controls (*n* = 376)	*P*-value
Age (mean ± SD)	28.8 ± 5.0	28.9 ± 4.9	N.S.
Primigravid (%)	55.5	46.0	N.S.
Primiparous (%)	70.3	59.3	N.S.
FH of hypertension (%)	28.9	14.6	0.02
Pre-pregnancy BMI ​ (mean ± SD)	22.4 ± 4.2	21.1 ± 3.0	<0.001
Gestational weeks ​ at delivery (mean ± SD)	36.6 ± 3.0	39.0 ± 1.2	<0.001
Birth weight (mean ± SD)	2421.1 ± 803.4	3114.7 ± 387.8	<0.001
IUGR (%)	33.5	3.2	<0.001

FH: family history

BMI: body mass index

IUGR: intrauterine growth retardation

N.S.: not significant

Table 2 shows the distribution of *PAI-1* polymorphisms among the 128 cases (101 patients with severe PE, 24 with severe PIH and delivery before or during gestational week 34, and 42 with severe PIH and delivery before or during gestational week 36) and the 376 controls with normal pregnancies. No significant differences were found between the cases and controls. Multivariate analysis revealed that a positive history of hypertension, the *AGT* TT genotype, the *NOS3* GA+AA genotype, and a pre-pregnancy BMI of 24 or higher were independently associated with severe PIH (Odds ratios (ORs): 2.53, 1.78, 2.21, and 2.44, respectively). On multivariate analysis, there was no significant interaction between the *PAI-1* 4G/4G genotype and either the *AGT* TT genotype or *NOS3* GA+AA genotype.

**Table 2.** Distribution of the *PAI-1* 4G/5G polymorphism among women with severe pregnancy-induced hypertension and healthy controls

Diagnosis	No. of cases	Genotype			Frequency of 4G Allele

		4G/4G (%)	4G/5G (%)	5G/5G (%)	
severe PIH	128	49 (33.3)	64 (50.0)	15 (11.7)	63.3%
severe PE^a^	101	37 (36.6)	52 (51.5)	12 (11.9)	62.3%
GW ≤34	24	9 (37.5)	11 (45.8)	4 (16.7)	60.4%
GW ≤36	42	16 (38.1)	22 (52.3)	4 (9.5)	64.3%
controls	376	144 (38.3)	178 (47.3)	54 (14.4)	62.0%

PIH: pregnancy-induced hypertension, PE: preeclampsia

^a^severe PE (severe proteinuric PIH) is included with severe PIH

GW: gestational weeks at delivery

Among the subgroup with the *PAI-1* 4G/4G genotype, the frequency of the *AGT* TT genotype was significantly higher among cases (77.6%) than among controls (56.9%; *P* < 0.0125); no significant difference was observed between cases and controls (70.8% vs. 61.2%) among the subgroup of subjects with either the *PAI-1* 5G/5G or 4G/5G genotypes (Table 3). The ORs and their 95% confidence intervals (95% CIs) among women with the *AGT* TT genotype and without the *PAI-1* 4G/4G genotype, those with the *PAI-1* 4G/4G genotype and without the *AGT* TT genotype, and those with both of these genotypes, as compared to women with neither of these genotypes, were 1.54 (0.89–2.68), 0.73 (0.33–1.61), and 1.84 (1.01–3.34), respectively. Multivariate analysis of risk factors, including parity, maternal age, FH of hypertension, high pre-pregnancy BMI, and *NOS3* GA+AA genotype, revealed that the *AGT* TT genotype, a positive FH of hypertension, and advanced maternal age were strong independent risk factors for severe PIH among the subgroup with the *PAI-1* 4G/4G genotype; the estimated ORs and their 95% CIs were 2.75 (1.22–6.17), 2.48 (1.10–5.56), and 2.87 (1.26–6.54), respectively.

**Table 3.** Distribution of the M235T allele of the angiotensinogen gene, by *PAI-1* genotype, among cases and controls

Diagnosis	No. of subjects	*AGT *genotype			Frequency of TT

		MM (%)	MT (%)	TT (%)	
4G/4G genotype of *PAI-1*					
cases	49	1 (2.0)	10 (20.4)	38 (77.6)	77.6%^a^
controls	144	6 (4.2)	56 (38.9)	81 (56.9)	56.9%

4G/5G + 5G/5G genotype of *PAI-1*					
cases	79	3 (3.8)	20 (25.3)	56 (70.8)	70.8%
controls	232	7 (3.0)	83 (35.8)	142 (61.2)	61.2%

^a^*P* < 0.0125 vs. controls

*PAI-1*: plasminogen activator inhibitor-1 gene

*AGT*: angiotensinogen gene

The frequency of the *NOS3* GA+AA genotype was significantly higher in cases (27.8%) than in controls (13.3%; *P* < 0.0125) among the subgroup with the *PAI-1* 5G/5G or 4G/5G genotypes; however, no significant difference was observed between cases and controls (18.4% vs 11.1%) among the subgroup with the *PAI-1* 4G/4G genotype (Table 4). The ORs and 95% CIs among women with the *NOS3* GA+AA genotype and without the *PAI-1* 5G/5G or 4G/5G genotypes, those with the *PAI-1* 5G/5G or 4G/5G genotypes and without the *NOS3* GA+AA genotype, and those with both of these genotypes, as compared to women with neither of these genotypes, were 1.80 (0.74–4.39), 0.91 (0.57–1.44), and 2.27 (1.18–4.36), respectively. Multivariate analysis revealed that the *NOS3* GA+AA genotype, a FH of hypertension, and high pre-pregnancy BMI were strong independent risk factors for severe PIH among the subgroup with the *PAI-1* 5G/5G or 4G/5G genotypes. The estimated ORs and 95% CIs were 2.51 (1.31–4.81), 2.86 (1.52–5.38), and 2.18 (1.11–4.24), respectively.

**Table 4.** Distribution of the Glu298Asp allele of the endothelial nitric oxide synthase gene, by *PAI-1* genotype, among cases and controls

Diagnosis	No. of cases	Genotype of *NOS3*			Frequency of GA+AA (%)

		GG (%)	GA (%)	AA (%)	
4G/4G genotype of *PAI-1*					
cases	49	40 (81.6)	9 (18.4)	0 (0.0)	18.4%
controls	144	128 (88.9)	16 (11.1)	0 (0.0)	11.1%

4G/5G + 5G/5G genotype of *PAI-1*					
cases	79	57 (72.2)	22 (27.8)	0 (0.0)	27.8%^a^
controls	232	201 (86.6)	30 (12.9)	1 (0.4)	13.3%

^a^*P* < 0.0125 vs. controls

*PAI-1*: plasminogen activator inhibitor-1 gene

*NOS3*: endothelial nitric oxide synthase gene

## DISCUSSION

In the present study, the distribution of *PAI-1* polymorphisms in cases and controls is concordant with those of control populations examined in other Japanese studies,^12^^,^^24^ and is also compatible with Hardy-Weinberg equilibrium. There was no significant association between the *PAI-1* 4G/4G genotype and severe PIH among pregnant Japanese women, possibly because the *PAI-1* 4G/5G phenotype is only weakly associated with severe PIH; however, people with the *PAI-1* 4G/4G genotype are reported to have an approximately 20% higher plasma PAI-1 level than those with other genotypes.^10^^,^^11^ In sum, these findings indicate that any possible association would depend on interactions with other genetic and environmental factors, which are potential confounders in studies of 4G/5G polymorphisms. Indeed, a number of factors, including pressor/depressor mechanisms, lipid metabolism, the intravascular system, placental dysfunction, and disorders of the nervous system, may be associated with PIH, as well as the blood coagulation system.

There were no differences between cases and controls regarding important characteristics such as maternal age, gravidity, and parity. There were also no differences regarding the severity of PIH or the prevalences of obesity and proteinurea (data not shown). Therefore, we conducted subgroup analyses of M235T of *AGT* and Glu298Asp of *NOS3* with respect to *PAI-1* genotype, in order to evaluate possible synergistic effects of these genotypes in the development of PIH. Furthermore, because classification of onset as early depends on genetic risk and inheritance,^25^ we described the distribution of PIH by gestation of 34 weeks or less and 36 weeks or less, as we lacked sufficient data on the week of PIH onset for all cases.

After adjustment for multiple comparison, a significant association with the *AGT* TT genotype remained only for the subgroup with the 4G/4G genotype, and a significant association with the *NOS3* GA+AA genotype remained only for the subgroup with the 4G/5G or 5G/5G genotypes (*P* < 0.0125 for both).

We previously demonstrated that the *AGT* TT genotype and the *NOS3* GA+AA genotype were strong independent risk factors for PIH, after adjusting for confounding factors on multivariate analysis.^17^ In addition, we found that the *AGT* TT genotype and pregnancy at an advanced maternal age were independently associated with PIH.^15^ Another study demonstrated that the *AGT* M235T polymorphism is in tight linkage disequilibrium with a molecular variant of the proximal promoter of *AGT*, which consists of an adenine instead of a guanine 6 nucleotides upstream from the site of transcription initiation (G-6A). In an in vitro study, -6A was associated with lower promoter activity than was -6G.^26^ T235 (-6A) is also reported to be associated with inadequate trophoblastic invasion of the uterine spiral arteries and narrowing of the spiral arterioles during early pregnancy, which can provoke the onset of PIH.^27^ We previously observed that, in women with the *AGT* TT genotype, mental stress during pregnancy was significantly associated with PIH.^28^

Because it enhances the vascular reactivity to an α1-adrenergic stimulant, the *NOS3* Asp298 variant has been thought to reduce stimulation of NO production in the endothelium, even though the Glu298Asp variant is not located in any functional consensus sequence.^29^ Findings from our previous association study suggested that *NOS3* Asp298, Val105 of GSTP1 gene (*GSTP1*), and pregnancy at an advanced maternal age were synergistically associated with the pathogenesis of PIH via reduction of NO production.^30^ Some experimental studies have reported that NOS inhibition by L-NG nitro arginine-methyl ester induces vascular PAI-1 expression, which is thought to occur during vascular injury^31^^,^^32^; however, the role of NO in the regulation of PAI-1 expression in humans is not well understood.

The findings from this study and previous studies suggest that severe PIH can occur when inhibition of fibrinolysis due to the *PAI-1* 4G/4G genotype coexists with decreased blood flow in the placenta, which is caused by undeveloped spiral arterioles resulting from the *AGT* TT genotype. However, among individuals with the *PAI-1* 4G/5G or 5G/5G genotypes, PAI-1 expression caused by decreased NO production in association with the *NOS3* GA+AA genotype may play a role in the onset of severe PIH.

The results of multivariate analyses suggest that there are at least 2 pathogenetic pathways in the development of severe PIH. One is the result of synergism between *AGT* TT, *PAI-1* 4G/4G, and other unidentified genetic factors associated with FH and/or aging; the other pathway results from synergism between the *NOS3* GA+AA genotype and other unidentified genetic factors associated with FH and/or obesity. This hypothesis concords with reports confirming an association of the *AGT* TT genotype with PIH rather than with essential hypertension.^33^^–^^36^ It is also consonant with reports of an association of *NOS3* GA+AA with PIH, atherosclerosis, and myocardial infarction.^23^^,^^37^^–^^39^

The present study does possess some limitations that should be taken into account, such as a lack of data on both serum PAI-1 concentration and the relation between lifestyle and the blood coagulation system. In addition, the study was limited to Japanese women, and the sample size was not large enough to definitively exclude the possibility of a negative association between PIH and the *PAI-1* 4G/4G genotype. Therefore, it will be necessary in the future to carry out confirmation studies in which the distribution of the *PAI-1* genotype is compared by *AGT* genotype and *NOS*3 genotype among a larger number of subjects. However, with respect to early prediction and prevention of severe PIH, although the *PAI-1* 4G/4G genotype alone was not a risk factor for severe PIH, the fact that *PAI-1* genotypes are associated with varying risks for severe PIH suggests that *PAI-1* genotyping of pregnant women, in combination with other factors, may be useful in the development of individualized measures that may prevent severe PIH.

In conclusion, we found no significant association between the *PAI-1* 4G/5G genotype and severe PIH in Japanese women. Subsequent subgroup analyses showed that the 4G/4G genotype and the 4G/5G or 5G/5G genotype of *PAI-1* may interact with the *AGT* TT genotype and the *NOS3* GA+AA genotype, respectively, in the pathogenesis of severe PIH.

## APPENDIX

Other Members of the Hokkaido Perinatal Epidemiology Study Group (Department of obstetrics and gynecology of Hokkaido University Hospital and its affiliated hospitals): Drs. Masaki Azuma, Emi H. Kato, Takayuki Koshida, Takayuki Kudo, Tsuyoshi Kusaka, Michiya Kuwabara, Keiichiro Sakai, Hirofumi Sato, Masashi Sogame, Norihiko Tsumura, Noriko Noro, Hitoshi Hareyama, and Tatsumi Yamaguchi.
